# Lactulose and Melibiose Attenuate MPTP-Induced Parkinson’s Disease in Mice by Inhibition of Oxidative Stress, Reduction of Neuroinflammation and Up-Regulation of Autophagy

**DOI:** 10.3389/fnagi.2020.00226

**Published:** 2020-07-24

**Authors:** Chih-Hsin Lin, Pei-Cih Wei, Chiung-Mei Chen, Yu-Ting Huang, Jia-Lan Lin, Yen-Shi Lo, Jia-Li Lin, Chung-Yin Lin, Yih-Ru Wu, Kuo-Hsuan Chang, Guey-Jen Lee-Chen

**Affiliations:** ^1^Department of Neurology, Chang-Gung Memorial Hospital, Chang-Gung University College of Medicine, Taoyuan, Taiwan; ^2^Taipei First Girls High School, Taipei, Taiwan; ^3^Medical Imaging Research Center, Institute for Radiological Research, Chang Gung University/Chang Gung Memorial Hospital, Taoyuan, Taiwan; ^4^Department of Life Science, National Taiwan Normal University, Taipei, Taiwan

**Keywords:** Parkinson’s disease, lactulose and melibiose, MPTP mice, oxidative stress, neuroinflammation, autophagy

## Abstract

Parkinson’s disease (PD) is a common neurodegenerative disease characterized by the progressive loss of dopaminergic (DAergic) neurons in the ventral brain. A disaccharide trehalose has demonstrated the potential to mitigate the DAergic loss in disease models for PD. However, trehalose is rapidly hydrolyzed into glucose by trehalase in the intestine, limiting its potential for clinical practice. Here, we investigated the neuroprotective potential of two trehalase-indigestible analogs, lactulose and melibiose, in sub-chronic 1-methyl-4-phenyl-1,2,3,6-tetrahydropyridine (MPTP)-induced mouse model of PD. Treatment with MPTP generated significant motor deficits, inhibited dopamine levels, and down-regulated dopamine transporter (DAT) in the striatum. Expression levels of genes involved in anti-oxidative stress pathways, including superoxide dismutase 2 (SOD2), nuclear factor erythroid 2-related factor 2 (NRF2), and NAD(P)H dehydrogenase (NQO1) were also down-regulated. Meanwhile, expression of the oxidative stress marker 4-hydroxynonenal (4-HNE) was up-regulated along with increased microglia and astrocyte reactivity in the ventral midbrain following MPTP treatment. MPTP also reduced the activity of autophagy, evaluated by the autophagosomal marker microtubule-associated protein 1 light chain 3 (LC3)-II. Lactulose and melibiose significantly rescued motor deficits, increased dopamine in the striatum, reduced microglia and astrocyte reactivity as well as decreased levels of 4-HNE. Furthermore, lactulose and melibiose up-regulated SOD2, NRF2, and NQO1 levels, as well as enhanced the LC3-II/LC3-I ratio in the ventral midbrain with MPTP treatment. Our findings indicate the potential of lactulose and melibiose to protect DAergic neurons in PD.

## Introduction

Parkinson’s disease (PD), characterized by resting tremor, rigidity, bradykinesia, and postural instability, is a common neurodegenerative disease in the elderly (Jankovic, 2008). The pathological studies find a massive loss of dopaminergic (DAergic) neurons in the pars compacta of the substantia nigra (Surmeier et al., 2017). The neurodegeneration of PD could be caused by a complex interaction of genetic and environmental factors (Kalia and Lang, 2015). Genetic mutations involved in the oxidative stress pathway, such as synuclein alpha (*SNCA*), parkin RBR E3 ubiquitin-protein ligase (*PRKN*), Parkinsonism associated deglycase (*DJ1*), PTEN induced kinase 1 (*PINK1*) and leucine-rich repeat kinase 2 (*LRRK2*), are reported in patients with familial PD (Dias et al., 2013; Zuo and Motherwell, 2013). Genetic variants in glucosylceramidase β (*GBA*), proved to be the main risk for developing PD (Murphy et al., 2014), affecting autophagy activities (Aharon-Peretz et al., 2004; Gan-Or et al., 2015). A variety of environmental insults, including pesticides and 1-methyl-4-phenyl-1,2,3,6-tetrahydropyridine (MPTP), specifically increase oxidative stress, damage DAergic neurons and produce parkinsonism similar to the main features to PD (Tuite and Krawczewski, 2007), although only prolonged chronic but not acute or sub-acute MPTP exposure in mice triggers the formation of α-synuclein inclusion pathology (reviewed in Konnova and Swanberg, 2018). Therefore, compounds that reduce oxidative stress and up-regulate autophagy may be therapeutic strategies for PD patients.

Trehalose, a disaccharide found in plants and animals, demonstrates the potential to assist protein folding during environmental stress (Elbein et al., 2003). In cell and rodent models of Alzheimer’s disease (AD), trehalose protects neurons by reducing aggregation of Aβ and could be a therapeutic candidate for AD (Liu et al., 2005; Du et al., 2013). Trehalose also demonstrates neuroprotective potential in other aggregation-prone neurodegenerative diseases such as Huntington’s disease (Tanaka et al., 2004), amyotrophic lateral sclerosis (Castillo et al., 2013) and spinocerebellar ataxia (SCA) type 17 (Chen et al., 2015). Neuroprotective and anti-neuroinflammatory effects of trehalose were also observed in a chronic MPTP-induced PD mouse model (Sarkar et al., 2014). Also, trehalose could accelerate the clearance of mutant huntingtin/α-synuclein (Sarkar et al., 2007), TATA-box binding protein (Lee et al., 2015) and ataxin 3 (Lin et al., 2016) by enhancement of autophagy. However, trehalose is rapidly hydrolyzed by trehalase in the intestine (Dahlqvist, 1968), limiting its application for disease treatment.

Previously two trehalase-indigestible analogs, lactulose, and melibiose were found to up-regulate autophagy in aggregation-associated SCA type 3 and 17 cell models (Lee et al., 2015; Lin et al., 2016). In the present study, we examined the neuroprotective potential of trehalose and these two disaccharides in the MPTP-induced PD mouse model. Our findings provide new drug candidates for PD *via* up-regulating anti-oxidative stress and autophagy pathways as well as reducing neuroinflammation.

## Materials and Methods

### Test Disaccharides

Trehalose and melibiose were obtained from Sigma–Aldrich Company (St. Louis, MO, USA). Lactulose was purchased from ACROS Organics (Geel, Belgium).

### Sub-chronic MPTP Mouse Model

The animal experiments were conducted following the guidelines and were approved by the National Taiwan Normal University (NTNU) Research Committee. Male C57BL/6 mice (8 weeks old, 18–22 g) were purchased from the National Laboratory Animal Center (Tainan City, Taiwan). The mice were kept in individually ventilated cages under controlled temperature (25 ± 2°C), relative humidity (50%), and 12 h on/off light cycle with *ad libitum* access to food and water at the Animal House Facility of NTNU.

**Figure 1:**
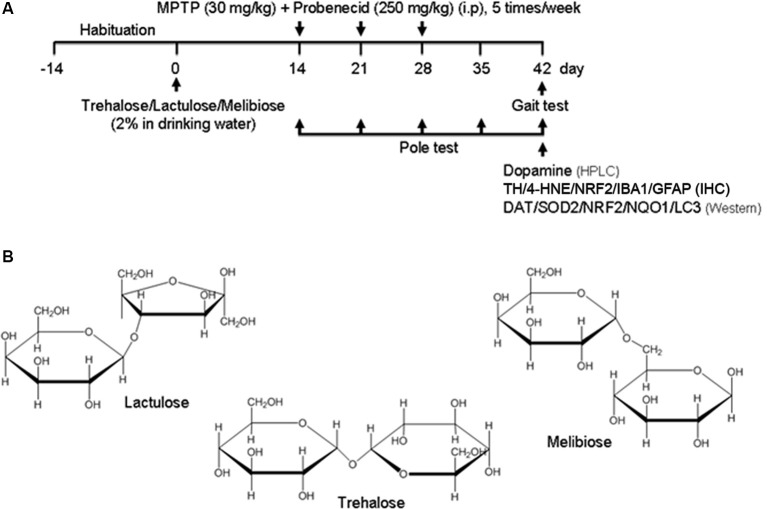
Sub-chronic 1-methyl-4-phenyl-1,2,3,6-tetrahydropyridine (MPTP) mouse model. **(A)** Experimental procotol. Parkinsonism was established by MPTP injections in C57BL/6 mice on the 14th day (15 dose regimen administered over 3 weeks) of 42-day duration of experiment. Mice received tested disaccharides from day 0 for 42 days. Saline-injected mice served as the control group. Pole test was performed on days 14, 21, 28, 35 and 42, and gait test was performed on day 42. Subsequently, mice were sacrificed for dopamine (by HPLC), tyrosine hydroxylase (TH), 4-hydroxynonenal (4-HNE), nuclear factor erythroid 2-related factor 2 (NRF2), ionized calcium-binding adapter molecule 1 (IBA1) and glial fibrillary acidic protein (GFAP; by IHC), and dopamine transporter (DAT), superoxide dismutase 2 (SOD2), NRF2, NQO1 and light chain 3 (LC3; by Western) analyses. **(B)** Structure of trehalose, lactulose and melibiose (formula C_12_H_22_O_11_, molar mass 342.30).

After 2-week habituation, mice were randomly divided into five groups (*n* = 8). Regular drinking water or drinking water with 2% trehalose, lactulose, or melibiose was applied to the mice for 42 days. Experimental parkinsonism was established by i.p. injections of 15 total doses of MPTP (30 mg/kg in 0.9% saline; Toronto Research Chemicals, Toronto, ON, Canada) along with probenecid (250 mg/kg in 0.1 M NaOH; Sigma–Aldrich), while the control group received injections of saline. Probenecid was administered 1 h before MPTP administration as it decreases the clearance of MPTP and intensifies its neurotoxicity (Lau et al., 1990). The 15 dose regimen was administered over 3 weeks with five doses per week (once daily for five consecutive days, see flow chart in Figure 1A). Appropriate guidelines were abided in handling MPTP. The water was changed once a week and mouse body weight, blood glucose, and drinking amount were monitored every week for 4 weeks. There was no notable difference in terms of mouse body weight, blood glucose, and drinking amount among these five groups. Behavioral analyses were performed during the period to evaluate the treatment effect.

### Behavioral Test

The pole test is a practical method to detect the degree of bradykinesia in the PD mouse model (Ogawa et al., 1985). Mice were placed head down on top of a vertical wooden pole (diameter 8 mm, height 50 cm), which was wrapped in gauze to prevent slipping (Yang et al., 2011). The time it took for the mice to climb down with all four feet on the floor was measured. Each mouse was required to perform three successive trials at 5 min interval. This test was performed at days 14, 21, 28, 35, and 42 (see flow chart). All the mice were pre-trained three times before the formal tests.

Also, stride length was measured in a gait test (Klapdor et al., 1997). To obtain footprints, the front and back paws were painted with nontoxic red and blue paints, respectively. Mice were allowed to walk along a narrow, paper-covered corridor (50 × 10 cm) toward a goal box, and stride length were measured manually as the distance between two paw prints using a digital vernier caliper. This test was performed on day 42, and the average of three strides was taken for each animal.

### HPLC Analysis of Dopamine

Levels of dopamine in the striatum were determined by high-performance liquid chromatography (HPLC) analysis. Briefly, the isolated brain striatum was homogenized in 500 μl of PRO-PREP™ protein extraction solution (iNtRON Biotechnology Inc., Gyeonggi-do, Korea). The samples were centrifuged at 10,000× *g* for 30 min and then filtered through a 0.45 μm syringe membrane. Dopamine from the supernatant was analyzed by the HPLC system using a C18 column with a UV detector at 254 nm. The sample was passed through the HPLC system using a mobile phase of 87.5% 90 mM of sodium phosphate, 40 mM of citric acid, 10 mM of octane sulfonic acid, 3 mM of ethylenediaminetetraacetic acid and 12.5% acetonitrile (pH 3.0) at a flow rate of 1.0 ml/min.

### Immunohistochemistry Analysis

Brains of mice were washed in PBS, fixed in 4% paraformaldehyde (PFA), cryoprotected in 30% sucrose in PBS, and embedded in optimal cutting temperature (OCT) compound before cryosectioning. Three 20-μm thick sections of midbrain were cut, washed twice with PBS, and fixed in 4% PFA in PBS for 20 min at room temperature. After two rinses with PBS + 0.2% Triton (PBST) for 5 min each, sections were blocked in PBST with 3% normal serum followed by incubation with tyrosine hydroxylase (TH; 1:50; MyBioSource, San Diego, CA, USA), 4-hydroxynonenal (4-HNE; 1:50; Cell Biolabs, San Diego, CA, USA), nuclear factor erythroid 2-related factor 2 (NRF2; 1:50; Boster Biological Technology, Pleasanton, CA, USA), ionized calcium-binding adapter molecule 1 (IBA1; 1:1,000; Wako, Osaka, Japan) or glial fibrillary acidic protein (GFAP; 1:1,000; Invitrogen, Waltham, MA, USA) primary antibody in blocking solution overnight at 4°C. After the incubation, cells were washed three times with PBST for 20 min and then incubated for 3 h with the secondary antibody (anti-goat or anti-rabbit IgG, 1:1,000; Invitrogen) in blocking solution in the dark. Sections were counterstained with 4′,6-diamidino-2-phenylindole (DAPI; 1:1,000; Enzo Life Sciences, Farmingdale, NY, USA) for 1 h. Quantitative analysis of TH, 4-HNE, NRF2, IBA1, or GFAP positive cells was carried out as the number of immune-positive cells with a clearly defined nucleus (identified by DAPI). MetaXpress software was applied for the determination of positive TH/4-HNE/NRF2/IBA1/GFAP cells. At least 500 cells were counted in each of the tested animals. The fluorescent intensities of IBA1 and GFAP were analyzed with ImageJ software (National Institutes of Health, ImageJ 1.40).

### Western Blot Analysis

The ventral midbrain was removed immediately after the mouse was sacrificed. The tissue was homogenized by Bullet Blender (Next Advance, Averill Park, NY, USA) with zirconium oxide grinding beads (1 mm; Next Advance) for 3 min in RIPA buffer (50 mM Tris-HCl pH 8.0, 150 mM NaCl, 1 mM EDTA, 1 mM EGTA, 1% NP-40, 0.5% sodium deoxycholate, 0.1% SDS) containing protease inhibitor (Sigma–Aldrich). The samples were incubated in ice for 30 min and then centrifuged at 15,000 *g* for 30 min at 4°C. The supernatant was collected and quantified by Bradford protein assay (Bio-Rad, Hercules, CA, USA). Proteins were separated on SDS-polyacrylamide gel electrophoresis and blotted on to polyvinylidene fluoride membranes (Pall Corporation, Port Washington, NY, USA) by reverse electrophoresis. After blocking, the membrane was probed with anti-dopamine transporter (DAT; 1:500; Santa Cruz Biotechnology, Santa Cruz, CA, USA), anti-superoxide dismutase 2 (SOD2; 1:500; Santa Cruz Biotechnology), anti-NRF2 (1:1,000; Boster Biological Techology), anti-NQO1 (NAD(P)H dehydrogenase, quinone 1; 1:1,000; Abcam, Cambridge, UK), anti-LC3 (microtubule-associated protein 1 light chain 3; 1:2,000; MBL international corporation, Woburn, MA, USA) or anti-GAPDH (glyceraldehyde-3-phosphate dehydrogenase; 1:5,000; MDBio, Taipei, Taiwan) at 4°C overnight. The immune complexes were subsequently detected by horseradish peroxidase-conjugated goat anti-rabbit IgG antibody (1:5,000; GeneTex, Irvine, CA, USA) and chemiluminescent substrate (Millipore, Billerica, MA, USA).

### Statistical Analysis

For each set of values, three independent experiments were performed and data were expressed as the means ± standard deviation (SD). Differences between groups were evaluated by student’s *t*-test or ANOVA followed by an LSD *post hoc* test where appropriate. All *P*-values were two-tailed, with values of *P* < 0.05 considered significant.

## Results

### Effects of Trehalose, Lactulose, and Melibiose on MPTP-Induced Motor Behavior in Mice

**Figure 2:**
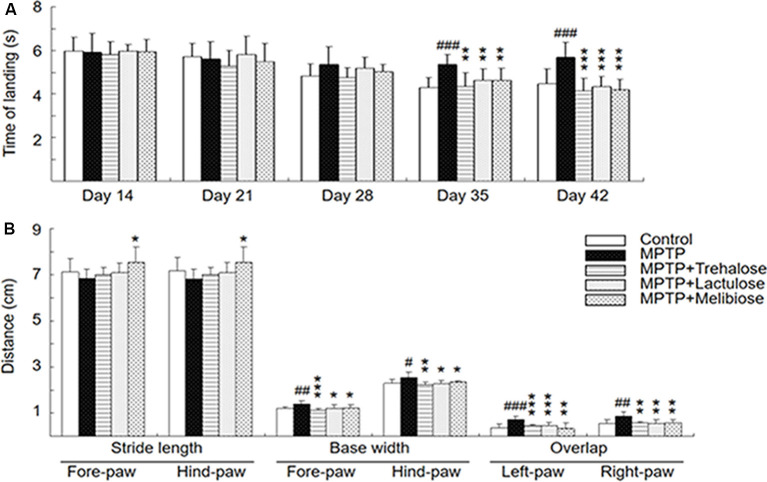
Neuroprotective effects of trehalose and analogs in the MPTP-induced Parkinson’s disease (PD) mouse model. **(A)** Pole test was conducted at days 14, 21, 28, 35 and 42. The time for mice to turn completely downward and land on the floor was recorded (*n* = 8). **(B)** Gait test was conducted at day 42. Stride length (fore-paw and hind-paw), base width (fore-paw and hind-paw) and overlap (left-paw and right-paw) were measured manually as the distance between two paw prints (*n* = 8). *P*-values, ANOVA with LSD *post hoc* test, MPTP vs. control (^#^*P* < 0.05, ^##^*P* < 0.01 and ^###^*P* < 0.001) and disaccharide-treated vs. untreated (*P < 0.05, **P < 0.01 and ***P < 0.001).

MPTP, a prodrug to the neurotoxin MPP^+^ which selectively destroys DAergic neurons in the brain, was frequently applied to establish a mouse model for PD (Blandini and Armentero, 2012). MPTP treatment in mice also down-regulated autophagy and increased the level of α-synuclein, while enhancement of autophagy reduced the loss of DAergic neurons (Liu et al., 2013). Given that trehalose could up-regulate autophagy and demonstrate neuroprotective potential in MPTP-treated mice (Sarkar et al., 2007, 2014), we established a sub-chronic MPTP mouse model (Figure 1A) to examine the neuroprotective effects of trehalose and its analogs lactulose and melibiose (Figure 1B) on PD. Trehalose is formed by a 1,1-glycosidic bond between two α-glucose units. Lactulose is a synthetic disaccharide comprising fructose and galactose. It is produced by the isomerization of lactose with chemical or enzymatic methods (Aider and de Halleux, 2007). Melibiose exists in natural plants such as cacao beans and is formed by an α-1, 6 linkage between galactose and glucose. In the pole test, before MPTP administration (day 14), there were no differences in the time of landing between the five groups (control group: 6.0 ± 0.6 s, MPTP group: 5.9 ± 0.9 s, trehalose-treated group: 5.8 ± 0.6 s, lactulose-treated group: 6.0 ± 0.3 s, and melibiose group: 5.9 ± 0.6 s; *P* > 0.05), indicating the presence of similar baselines for all groups (Figure 2A). After neurotoxin injection, MPTP-treated mice showed a marked motor deficit (24–27% increase of landing time) as compared with the control group (5.4 ± 0.4 s vs. 4.3 ± 0.5 s at day 35, 5.7 ± 0.7 s vs. 4.5 ± 0.7 s at day 42; *P* < 0.001). On the other hand, mice with trehalose treatment displayed recovery (4.3 ± 0.7 s at day 35, *P* < 0.01; 4.1 ± 0.6 s at day 42, *P* < 0.001) in comparison to mice with MPTP only. Moreover, treatment of lactulose or melibiose also exhibited significant improvement on landing time (decrease of time to reach the floor: 13% at day 35, *P* < 0.01; 24–26% at day 42, *P* < 0.001).

On the gait test, MPTP injection led to a shorter stride length at day 42 compared to the normal control (fore-paw: 6.8 ± 0.4 vs. 7.1 ± 0.6 cm; hind-paw: 6.8 ± 0.4 vs. 7.2 ± 0.6 cm; *P* > 0.05; Figure 2B). Although not significant, there was also a trend toward improving gait distance for both fore-paw and hind-paw in the trehalose (7.0 ± 0.3 cm) and lactulose (7.1 ± 0.4 cm)-treated groups (*P* > 0.05) compared to the MPTP only group. However, treatment with melibiose markedly prevented the decrease of the stride length (7.5 ± 0.7 cm for both fore-paw and hind-paw, *P* < 0.05). For base width, MPTP injection led to a significant increase compared to the normal control (fore-paw: 1.4 ± 0.1 vs. 1.2 ± 0.1 cm, *P* < 0.01; hind-paw: 2.5 ± 0.2 vs. 2.3 ± 0.1 cm, *P* < 0.05; Figure 2B). Treatment with trehalose, lactulose and melibiose markedly decreased base width for both fore-paw (1.1 ± 0.1 cm for trehalose, *P* < 0.01; 1.2 ± 0.1 cm for lactulose, *P* < 0.05; 1.2 ± 0.1 cm for melibiose, *P* < 0.05) and hind-paw (2.2 ± 0.1 cm for trehalose, *P* < 0.01; 2.3 ± 0.2 cm for lactulose, *P* < 0.05; 2.4 ± 0.1 cm for melibiose, *P* < 0.05). Similar trends of stride overlap in left-paw and right-paw with MPTP injection (increase of stride overlap: right-paw, *P* < 0.01; left-paw, *P* < 0.001) and disaccharide treatment (decrease of stride overlap, right-paw, *P* < 0.01; left-paw, *P* < 0.001) were also observed (Figure 2B). Thus, through pole and gait tests, neuroprotective effects of trehalose, lactulose and melibiose were observed in sub-chronic MPTP-induced PD mouse model.

### Effects of Trehalose, Lactulose and Melibiose on Dopamine, TH, DAT, SOD2 and 4-HNE Levels in MPTP-Treated Mice

**Figure 3:**
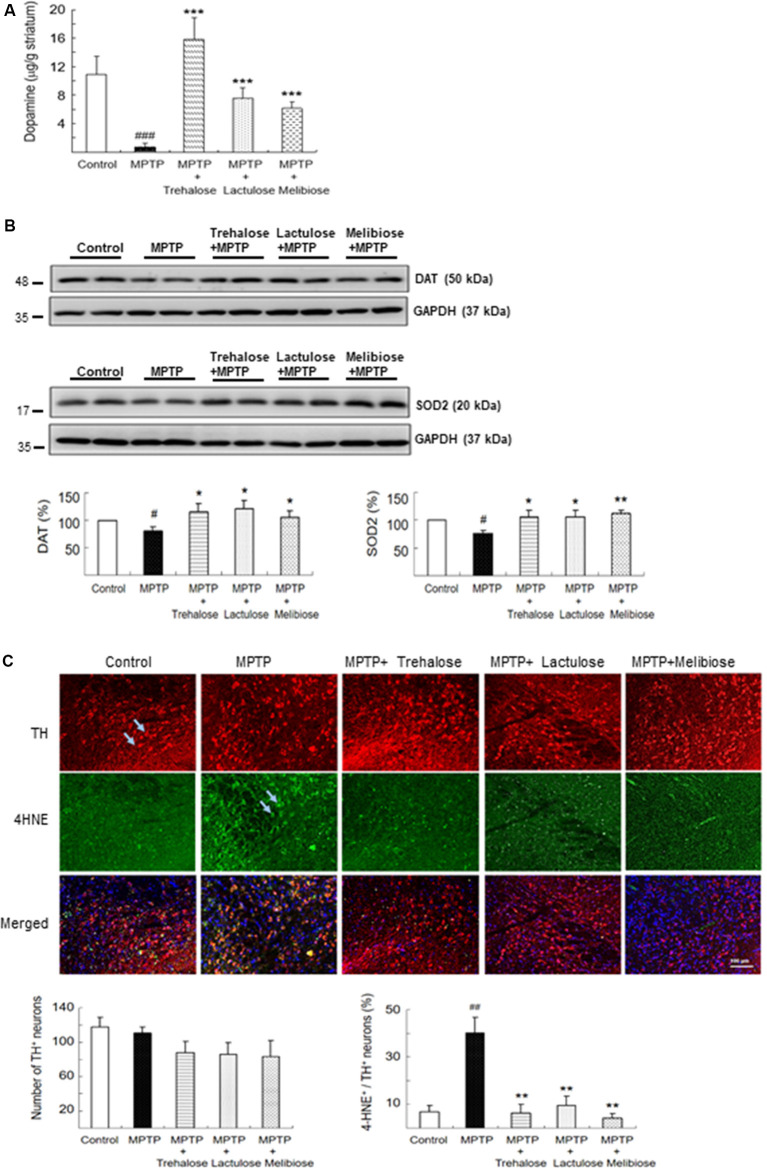
Effects of trehalose and analogs in dopamine secretion and oxidative damage on dopaminergic neurons. **(A)** Relative levels of dopamine determined by HPLC assay in mouse striatum (*n* = 8). **(B)** Western blotting to examine altered protein levels of DAT and SOD2 in ventral midbrain (*n* = 8, divided into four batches). **(C)** Immunohistochemistry of TH (red) and 4-HNE (green) positive neurons in ventral midbrain with MPTP/trehalose/lactulose/melibiose treatment. Nuclei were counter stained with 4^′^,6-diamidino-2-phenylindole (DAPI; blue). Percentage of dopaminergic neurons with oxidative damages, based on TH and 4-HNE co-localization, were shown below (*n* = 8). *P*-values, ANOVA with LSD *post hoc* test, MPTP vs. control (^#^*P* < 0.05, ^##^*P* < 0.01 and ^###^*P* < 0.001) and disaccharide-treated vs. untreated (*P < 0.05, **P < 0.01 and ***P < 0.001).

In mice, MPTP treatment promotes the formation of reactive free radicals and the reduction of dopamine production (Blandini and Armentero, 2012). By examining the dopamine levels of the striatum with HPLC, we consistently found that administration of MPTP significantly reduced dopamine levels (0.66 ± 0.61 μg/g tissue, *P* < 0.001) compared with controls (10.95 ± 2.44 μg/g tissue), while treatment with trehalose (15.85 ± 2.96 μg/g tissue, *P* < 0.001), lactulose (7.61 ± 1.43 μg/g tissue, *P* < 0.001) and melibiose (6.14 ± 0.91 μg/g tissue, *P* < 0.001) successfully rescued the reduction of striatal dopamine level caused by MPTP (Figure 3A). Interestingly, treatment with trehalose improved striatal dopamine levels greater than that of lactulose (*P* < 0.001) and melibiose (*P* < 0.001). In addition, MPTP administration significantly reduced DAT (80%, *P* < 0.05) and SOD2 (77%, *P* < 0.05) levels, and treatment with lactulose and melibiose successfully rescued the reduction of DAT (106–121% vs. 80%, *P* < 0.05) and SOD2 (106% vs. 77%, *P* < 0.05; 112% vs. 77%, *P* < 0.01) levels in the ventral midbrain (Figure 3B). Although the number of TH^+^ neurons was not significantly changed by MPTP and/or trehalose/lactulose/melibiose treatment, administration of MPTP significantly up-regulated the oxidative stress marker 4-HNE in TH^+^ neurons in the ventral midbrain (from 7% to 40%, *P* < 0.01), while treatment with trehalose, lactulose, and melibiose successfully rescued the up-regulation of 4-HNE in TH^+^ neurons (4–10% vs. 40%, *P* < 0.01; Figure 3C). Consistent with other studies (Fornai et al., 2005; Konnova and Swanberg, 2018), we did not find any intracellular inclusions immunoreactive for α-synuclein (data not shown). These results suggest the potential of trehalose, lactulose, and melibiose in ameliorating MPTP-induced damage on DAergic neurons in the ventral midbrain and the capacity to recover dopamine levels in the striatum.

### Enhancement of Autophagy, Anti-oxidant Stress Components, and Reduction of Neuroinflammation by Trehalose, Lactulose, and Melibiose in MPTP-Treated Mice

**Figure 4:**
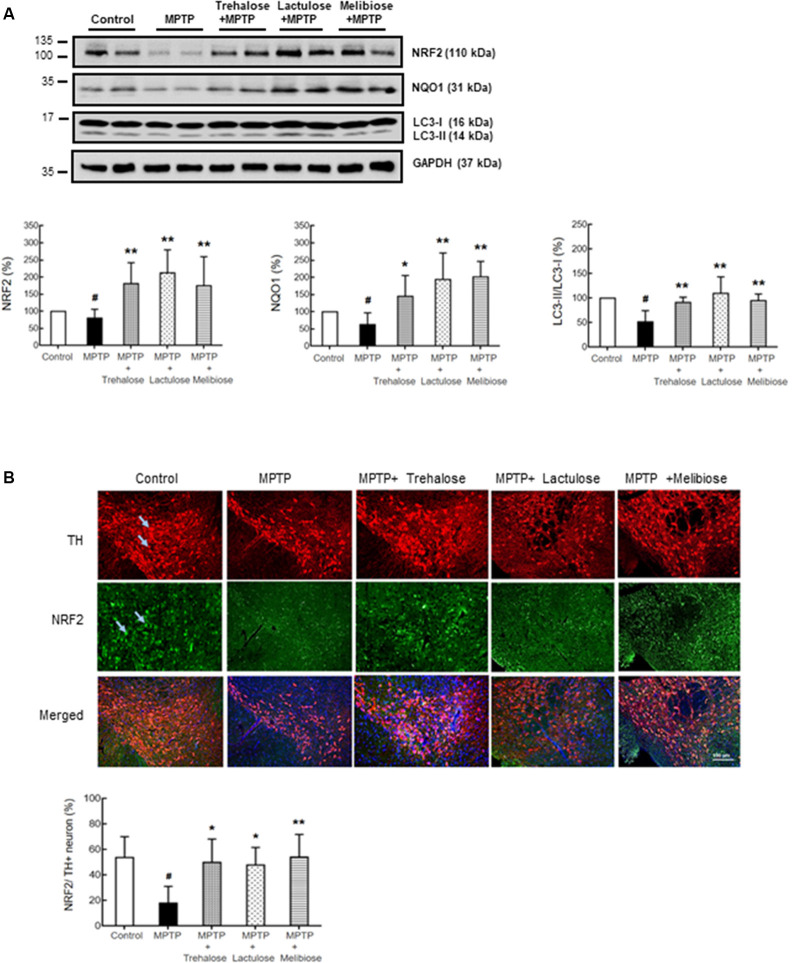
Trehalose and both analogs enhanced autophagy and decreased oxidative stress on dopaminergic neurons. **(A)** Western blotting to examine the altered protein levels of NRF2, NQO1 and LC3-II/I in ventral midbrain (*n* = 8, divided into four batches). **(B)** Immunohistochemistry of TH (red) and NRF2 (green) positive neurons in ventral midbrain with MPTP/trehalose/lactulose/melibiose treatment. Nuclei were counter stained with DAPI (blue). Percentages of dopaminergic neurons with anti-oxidative damage, identified by TH and NRF2 co-localization, were shown below (*n* = 8). *P*-values, ANOVA with LSD *post hoc* test, MPTP vs. control (^#^*P* < 0.05) and disaccharide-treated vs. untreated (*P < 0.05 and **P < 0.01).

**Figure 5:**
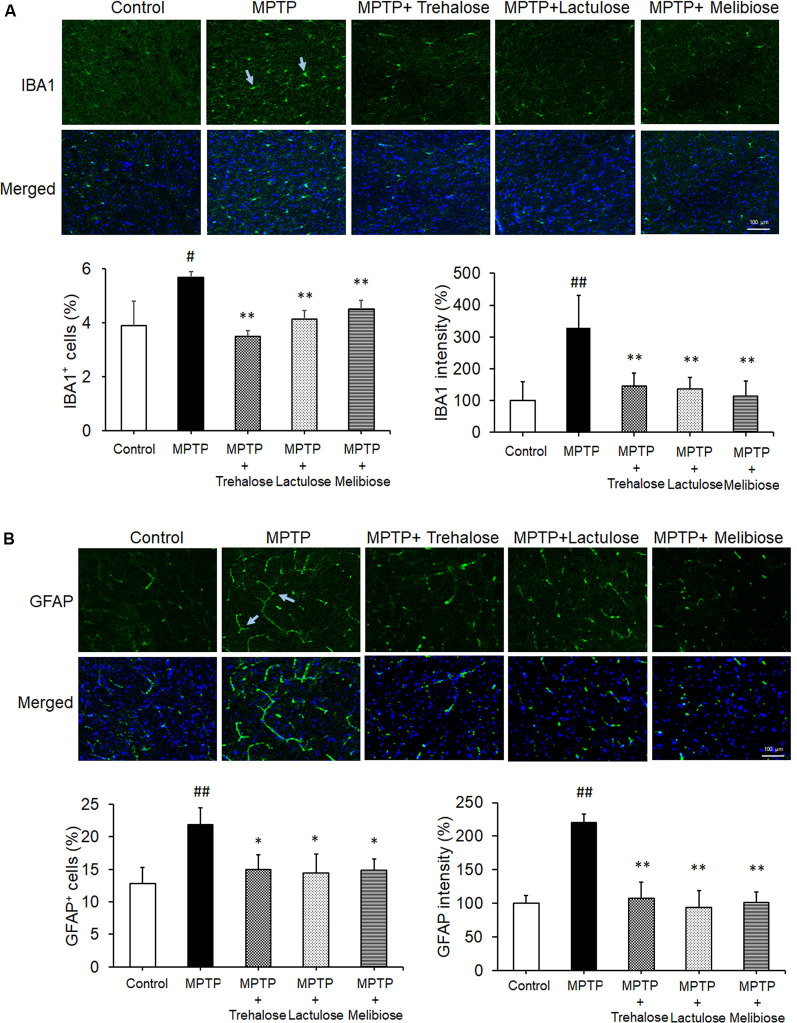
Trehalose and both analogs reduced neuroinflammation in the ventral midbrain. **(A)** Immunohistochemistry of IBA1 positive microglia in the ventral midbrain with MPTP/trehalose/lactulose/melibiose treatment. Nuclei were counter stained with DAPI (blue). Percentages of IBA1^+^ cells and fluorescent intensity of IBA1 were shown below (*n* = 8). **(B)** Immunohistochemistry of GFAP positive astrocytes in the ventral midbrain with MPTP/trehalose/lactulose/melibiose treatment. Nuclei were counter stained with DAPI (blue). Percentages of GFAP^+^ cells and fluorescent intensity of GFAP were shown below (*n* = 8). *P*-values, ANOVA with LSD *post hoc* test, MPTP vs. control (^#^*P* < 0.05 and ^##^*P* < 0.01) and disaccharide-treated vs. untreated (^*^P < 0.05 and ^**^P < 0.01).

We further investigated the potential effects of trehalose, lactulose and melibiose on anti-oxidative stress and autophagic pathways, as well as anti-neuroinflammation by examining the expression levels of NRF2 and NQO1 (anti-oxidative markers), LC3 (autophagic marker), IBA1 (microglial activation marker) and GFAP (astrocyte activation marker) in the ventral midbrain. Treatment with trehalose, lactulose and melibiose significantly rescued the down-regulation of NRF2 (trehalose: 180%, *P* < 0.05; lactulose: 212%, *P* < 0.01; melibiose: 174%, *P* < 0.05) and NQO1 (trehalose: 145%, *P* < 0.05; lactulose: 193%, melibiose: 201%, *P* < 0.01) in the ventral midbrain of mice treated with MPTP (MPTP only: NRF2: 80%, NQO1: 63%, *P* < 0.05; Figure 4A). The immunohistochemical study consistently showed that NRF2 in TH-positive DAergic neurons in the ventral midbrain was down-regulated by MPTP (19%, *P* < 0.01), while treatment with trehalose/lactulose/melibiose rescued this down-regulation (trehalose: 50%, lactulose: 47%, melibiose: 54%, *P* < 0.01; Figure 4B). In the ventral midbrain, the LC3-II/I ratio, an indicator of autophagy activity, was reduced by MPTP (52%, *P* < 0.01), while treatment with trehalose/lactulose/melibiose rescued this reduction of LC3-II/I ratio (trehalose: 91%, lactulose: 110%, melibiose: 95%, *P* < 0.01; Figure 4A). MPTP increased the percentage of IBA1^+^ microglia (from 3.9% to 5.7%, *P* < 0.05), while treatment with trehalose/lactulose/melibiose reduced this microglial activation (trehalose: 3.5%, lactulose: 4.1%, melibiose: 4.5%, *P* < 0.01; Figure 5A). Consistently, IBA1 fluorescent intensity was up-regulated by MPTP treatment (328%, *P* < 0.01), while treatment with trehalose/lactulose/melibiose reduced IBA1 fluorescent intensity (trehalose: 145%, lactulose: 136%, melibiose: 113%, *P* < 0.01). The percentage of GFAP^+^ astrocytes was increased by MPTP treatment (from 12.8% to 21.9%, *P* < 0.01). Treatment with trehalose/lactulose/melibiose reduced the percentage of GFAP^+^ astrocytes (trehalose: 15.0%, lactulose: 14.5%, melibiose: 14.9%, *P* < 0.05; Figure 5B). GFAP fluorescent intensity was also up-regulated by MPTP treatment (221%, *P* < 0.01), while treatment with trehalose/lactulose/melibiose reduced fluorescent intensity of GFAP (trehalose: 107%, lactulose: 97%, melibiose: 101%, *P* < 0.01). Taken together, trehalose, lactulose and melibiose improved the down-regulation of anti-oxidative stress pathways and autophagy activity, as well as decreased neuroinflammation induced by MPTP.

## Discussion

Increased oxidative stress and decreased antioxidant capacity including reduced SOD and increased 4-HNE are among pathological findings in postmortem brains of human PD and the MPTP-induced PD mouse model (Yoritaka et al., 1996; Castellani et al., 2002; Sofic et al., 2006; Li and Pu, 2011; Lv et al., 2012). Recently, *in vitro* studies showed that treatment with trehalose significantly reduced oxidative stress induced by chloroquine or cadmium *via* activating the NRF2 pathway, suggesting its strong anti-oxidant effect (Mizunoe et al., 2018; Wang et al., 2018). It is important to note that trehalose is readily digested by trehalase in the gut of humans (Dahlqvist, 1968), which implicates trehalase-indigestible analogs rather than trehalose as the potential treatments for aggregation-associated neurodegenerative disease. Here, we demonstrated the anti-oxidative and neuroprotective effects of two trehalase-indigestible analogs, lactulose, and melibiose, in the MPTP-induced PD mouse model. Although the elevations of striatal dopamine levels by lactulose and melibiose may be lower compared with trehalose, both of them still demonstrate improvements of motor deficits similar to trehalose. Furthermore, lactulose and melibiose increased DAT, SOD2, NRF2, and NQO1, and decreased 4-HNE, IBA1, and GFAP in the ventral midbrain of MPTP-induced PD mice. These findings suggest that lactulose and melibiose, similar to trehalose, may exert their anti-oxidative and anti-neuroinflammatory capacity to provide neuroprotection. Consistent with our findings, Sarkar et al. (2014) also demonstrate that trehalose can reduce the activation of microglia and astrocytes in the MPTP-induced PD mouse model.

Lines of evidence implicate targeting autophagy as a potential PD therapeutic strategy (Moors et al., 2017; Zhu et al., 2019). The depletion of autophagy gives rise to neurotoxicity accumulation and causes the loss of nerve cells (Hara et al., 2006; Komatsu et al., 2006). It has been proved that α-synuclein fibrils or aggregates are cleared by the autophagy-lysosomal pathways (Bae et al., 2014). Moreover, PD-associated proteins including LRRK2 (Orenstein et al., 2013; Manzoni et al., 2016), PINK1 (Lazarou et al., 2015), PRKN (Narendra et al., 2008) and ATP13A2 (ATPase cation transporting 13A2; Bento et al., 2016) are involved in autophagy-processing modulation as well. As an autophagy inducer, trehalose has the therapeutic potential on cellular and animal models of aggregation-prone neurodegenerative diseases (Sarkar et al., 2007; Rodríguez-Navarro et al., 2010; Casarejos et al., 2011; Lan et al., 2012; Schaeffer et al., 2012; Lee et al., 2015; Lin et al., 2016). In SCA17 and SCA3 cell models, we found that lactulose and melibiose demonstrate anti-aggregation and neuroprotection effects mainly through autophagy-activation (Lee et al., 2015; Lin et al., 2016). Our results showed MPTP treatment down-regulated autophagy function by reducing the conversion of LC3-II from LC3-I. Similar to trehalose, lactulose and melibiose increased the ratio of LC3-II/LC3-I in the ventral midbrain of MPTP-treated mice, suggesting their potential to up-regulate autophagy in PD.

This study demonstrates the neuroprotective potential of lactulose and melibiose in the MPTP-induced PD mouse model, by activating NRF2 and autophagy pathways. However, their neuroprotective effects may not be better than trehalose, even though they are trehalase-indigestible. Although not broken down by human enzymes, lactulose and melibiose can be metabolized in the colon by *Bifidobacterium*, *Lactobacillus* or *Saccharomyces* species (Ostergaard et al., 2000; Bouhnik et al., 2004; De Souza Oliveira et al., 2011), which may lead to less concentration of lactulose and melibiose in the brain. Further investigations to refine their metabolism by intestinal flora of microorganisms will be necessary to enhance their neuroprotective effects.

In conclusion, our results show that lactulose and melibiose reduce motor deficits, inhibit the loss of striatal dopamine, increase DAT, decrease 4-HNE level, reduce activation of microglia and astrocytes, and enhance anti-oxidative and autophagy functions in the ventral brain of MPTP-induced PD mice. Future studies in different PD models will be warranted to confirm their potentials as treatments for human PD.

## Data Availability Statement

All datasets generated for this study are included in the article.

## Ethics Statement

The animal study was reviewed and approved by National Taiwan Normal University (NTNU) Research Committee.

## Author Contributions

C-HL and P-CW: execution of experiments, data analysis and interpretation, and wrote the article. Y-TH, J-LanL, Y-SL, J-LiL and C-YL: execution of experiments. Y-RW: concept design and data analysis and interpretation. C-MC, K-HC, and G-JL-C: concept and design, data analysis and interpretation, obtained funding, wrote and finalized the article.

## Conflict of Interest

The authors declare that the research was conducted in the absence of any commercial or financial relationships that could be construed as a potential conflict of interest.
